# The Diagnostic Value of Serum Amyloid A and Other Laboratory and Clinical Variables in Cats with Increased Liver Enzyme Activity

**DOI:** 10.3390/vetsci11070298

**Published:** 2024-07-01

**Authors:** Josefine Öberg, Jens Häggström, Lena Pelander, Anna Hillström, Ingrid Ljungvall

**Affiliations:** 1 AniCura Bagarmossen Animal Hospital, 128 48 Stockholm, Sweden; 2 Department of Clinical Sciences, Faculty of Veterinary Medicine and Animal Science, The Swedish University of Agricultural Science, 750 07 Uppsala, Sweden; jens.haggstrom@slu.se (J.H.); lena.pelander@slu.se (L.P.); ingrid.ljungvall@slu.se (I.L.); 3 The University Animal Hospital, The Swedish University of Agricultural Science, 750 07 Uppsala, Sweden; anna.hillstrom@slu.se

**Keywords:** feline, hepatic, inflammation, lipidosis, acute phase proteins, SAA

## Abstract

**Simple Summary:**

In this retrospective medical record study, we evaluated if certain diagnostic variables, including Serum Amyloid A (SAA), could differentiate (1) between various clinical disease categories and (2) between cytological findings of severe hepatic lipidosis and other cytological findings in cats diagnosed with increased liver enzymes at a Swedish animal hospital. Grouping into four clinical disease categories (primary liver diseases, trauma, extrahepatic diseases, and other non-specified diagnoses) was based on clinical diagnosis or information from medical records. Serum Amyloid A was found to be higher in the group of cats with diagnoses supporting trauma. Cats with cytological findings supporting severe hepatic lipidosis had lower SAA and were younger, compared to cats with other cytological findings.

**Abstract:**

Distinguishing inflammatory from non-inflammatory liver disease in cats may impact management. The study aim was to evaluate if certain diagnostic variables, including Serum Amyloid A (SAA), differ (1) between various clinical disease categories (*Primary liver disease*, *Extrahepatic*, *Trauma* and *Inconclusive*) and (2) between cytological findings of severe hepatic lipidosis and other cytological findings in cats with increased liver enzymes. Medical records from 5042 cats, where SAA had been measured, were reviewed, and 566 cats fulfilled inclusion criteria consisting of increased liver enzymes and available biochemical panel results. SAA was higher in cats diagnosed with trauma compared to other diseases (*p* = 0.008). Cytology results were available in 85 cats, and cats with severe lipidosis had lower serum SAA concentration (*p* < 0.0001) and were younger (*p* < 0.0002) compared to cats with other cytological findings. The study shows that SAA was higher in cats diagnosed with trauma compared to cats with other causes of increased liver enzymes and that SAA may be useful to distinguish cats with cytologic evidence of hepatic lipidosis from cats with other liver pathologies. Serum Amyloid A may be a valuable complement to liver cytology when investigating cats with increased liver enzymes.

## 1. Introduction

Activity of the liver enzymes alanine amino transferase (ALT) and alkaline phosphatase (ALP) are commonly measured in blood samples from cats to detect and monitor diseases affecting the liver and/or biliary systems [1,2]. Increased liver enzyme activity in the blood can be caused by primary parenchymal liver cell damage, biliary stasis/obstruction, trauma to the liver, liver hypoxia, neoplastic diseases, and several endocrine disorders [1,2,3,4,5].

Depending on the cause and type of disease affecting the liver or biliary system, treatment regimens differ. Cats with hepatic lipidosis, a metabolic disorder with an energy imbalance, require urgent and aggressive feeding support, but antimicrobial therapy is not recommended [3,4,5,6]. To the contrary, antimicrobial therapy is recommended for cats with hepatobiliary inflammatory disorders with a septic component [7,8,9]. These divergent treatment recommendations illustrate a need for identification of a correct diagnosis. Because increased liver enzyme activity in a blood sample is an unspecific finding, additional diagnostic tests are usually required in order to reach a diagnosis. Samples from the liver and/or gall bladder can generate information about the type of liver disease [10,11,12,13,14,15]. The drawback of sampling from the liver and biliary system is the requirement of sedation or general anesthetic care and the risk of bleeding from the liver [4,16,17,18,19,20,21]. Furthermore, samples might have to be sent to external laboratories for analysis, leading to an increased turnaround time for the results. Due to above-mentioned reasons, tissue or cell sampling may deliberately be avoided, and information required for instituting adequate treatments may therefore be missing [14,15,16,17,18,19,20,21,22]. As a result, there is a risk of incorrect use of antimicrobial therapy or delayed optimal feeding support for diseased cats.

Analysis of acute phase proteins (APPs) is commonly used to detect systemic inflammatory diseases and for monitoring response to treatment [23,24,25,26]. In cats, the APP serum amyloid A (SAA) has been shown to be a valuable analyte for diagnosing and monitoring cats with different inflammatory diseases [24,27,28,29]. The additional value of SAA to other biomarkers for distinguishing cats with inflammatory liver disease from cats with non-inflammatory liver disease, such as hepatic lipidosis has, to our knowledge, not previously been investigated.

The aim of this retrospective study was to evaluate if cat characteristics, case history, physical examination findings, and laboratory variables, including SAA, differ (1) between clinical disease categories *(Primary liver disease*, *Trauma*, *Extrahepatic* and *Inconclusive*) and (2) between cytological findings of *Severe hepatic lipidosis* and *Other cytological findings* in a population of cats with increased serum liver enzyme activity examined at an animal hospital in Sweden.

## 2. Materials and Methods

### 2.1. Data Collection from Medical Records

The retrospective study was conducted at AniCura Bagarmossen small animal hospital in Sweden. Due to the retrospective nature of the study, no ethical approval was needed. A diagnostic index in the medical record system Trofast (version 8.6.0.8, Trofast AB, Västerås, Sweden) was used to search the database for cats that had visited the animal hospital between 1 January 2017 and 31 December 2020, and for which SAA had been analyzed. The medical records for these cats were reviewed by a board-certified clinical pathologist (JÖ). Cats with increase in ALT and/or ALP enzyme activity by two times or more from the upper limit of the reference values were selected for the study [30]. To be included in the study, results from a chemistry panel had to be available. Samples for chemistry analysis must have been taken at the same visit, as sampling and analysis for SAA was performed. In addition to ALT and ALP, the chemistry panel had to include, at a minimum, glucose, albumin, total protein, urea, and creatinine. If blood had been sampled and analyzed several times during the hospital visit, results from the first sampling were used in the study.

### 2.2. Blood Sample Routines and Laboratory Instruments Used at the Animal Hospital

Samples collected during the daytime were handled by trained biomedical analysts, and samples collected during night shifts were handled by specially trained nurses. All samples were analyzed using in-house validated instruments. Hematology was analyzed in EDTA-blood with a Sysmex XT-2000 iv (Sysmex Corporation, Norderstedt, Germany). Manual differential leukocyte counts were performed by trained biomedical analysts. This was not mandatory for inclusion in the study. Blood samples were stored in a refrigerator (6–7 °C) for a maximum of 24 h if not analyzed at the time of sampling, and a blood smear for differential counts was prepared before storage. Manual differential and morphology evaluation, for example for the presence and degree of toxicity, was performed by trained biomedical analysts. During the daytime, blood chemistry was analyzed in serum or heparin plasma with a Cobas c311 system (Cobas c311, Roche Diagnostics International AG, Rotkreuz, Switzerland). Cats sampled during night shifts had blood chemistry analyzed in heparin plasma with a Catalyst DX system (IDEXX Laboratories Inc., Westbrook, Main, USA), or serum was separated after centrifugation and stored in a refrigerator (6–7 °C) for analysis the next day using a Cobas c311 system. The reference intervals used for results from the Cobas c311 and Sysmex XT-2000 iv systems were established at the laboratory, mainly generated by transference studies. For the Catalyst DX, reference intervals given by the producer were used (IDEXX corporation). For samples analyzed with Cobas, the upper reference limit was 1.2 ukat/L for ALT and 1.0 ukat/L for ALP. For samples analyzed with Catalyst, the upper reference limit was 130 U/L for ALT and 110 U/L for ALP. All reference values for chemistry analytes used in the study are presented in Supplementary File, Table S1.

Serum amyloid A was analyzed with a human turbidimetric immunoassay (SAA-TIA; LZ-SAA, Eiken Chemical Co., Tokyo, Japan), previously validated for use in cats [31]. Analyses were performed on an automated analyzer (Cobas c311, Roche Diagnostics International AG, Rotkreuz, Switzerland). An in-house validation of the assay was performed. The measurement range was 10–100 mg/L, as determined by studies of linearity upon dilution. Intra- and inter-assay variation was 2.4% and 2.6%, respectively. Values below 10 mg/L were reported as <10 mg/L, and values above 100 mg/L were reported as >100 mg/L.

Semi-quantitative aerobic and anaerobic bacterial cultures from the bile were performed according to the routines at the laboratory.

### 2.3. Database

The following information from the medical records was entered into a data sheet (Microsoft Excel 365): (1) cat characteristics—age, sex, breed, body weight (BW), BW reduction (noted as decrease in body weight between consultation times or due to information from owner; yes or no, y/n); (2) case history—appetite (increased or decreased), presence of vomiting, and/or diarrhea (y/n), presence of anorexia (y/n), and if anorexia was noted, days of anorexia; (3) physical examination findings—body condition score (BCS) and body temperature; (4) laboratory variables—SAA, hematology profile, ALT, ALP, GGT, albumin, protein, glucose, urea, creatinine, cholesterol, bilirubin, bile acids, fine-needle aspiration (FNA) for cytology from liver and/or biliary system (y/n), and if cytology had been performed (yes), summary of the results, and microbiological culturing results; and (5) therapeutic intervention and clinical outcome—treatment protocol used involving antimicrobial therapy (y/n), feeding tube (y/n), and days of hospitalization. As different methods were used for chemistry depending on the time of day for analysis, results from blood biochemistry analysis were reported as above upper limit of refence range, within reference range, or below lower limit of reference range for all variables except for SAA, which was analyzed with one method in all cats, for which numerical values were used.

### 2.4. Clinical Diagnosis Categories

All cats included in the study were divided into four categories based on clinical diagnosis. Diagnoses in the medical records, which were established by the attending veterinarian at the time of the hospital visit according to an existing medical record system in Sweden, were used. If a diagnosis was missing, the medical record of the individual cat was critically reviewed by two of the authors, one board-certified internist (LP) and one board-certified clinical pathologist (JÖ), in order to, if possible, establish a diagnosis based on available information. Depending on the diagnosis, cats were allocated into the *Primary liver disease* category (*Primary liver*), *Trauma* category (*Trauma*), or *Extrahepatic disease* category (*Extrahepatic*). Cats with unspecific diagnoses, and for which case history and diagnostic information in the medical record could not be used to establish the cause of increase in liver enzymes, were included in the *Inconclusive* category (*Inconclusive*). Examples of diagnoses used to divide the cats into the different diagnosis-based categories are shown in Supplementary Files Table S2.

### 2.5. Cytology Groups

Cats for which cytological evaluation of the liver and/or bile was performed were identified and selected for further investigations. They were divided into two groups based on cytological interpretation (see below). Fine-needle aspiration (FNA) was performed during ultrasound examination with the cat sedated. Ultrasound examination and FNA was performed by trained diagnostic imaging veterinarians using the packing technique for liver aspiration and the routine aspiration technique for bile aspiration. Cytology evaluation was performed by one board-certified clinical pathologist (JÖ). Information collected from cytology reports in the medical records for liver cytology included degree of cellularity, proportion of hepatocytes with cytoplasmic vacuolation, amount of vacuolation in the cytoplasm, number of leukocytes present, and presence of atypical cells. The information collected from cats for which bile aspiration was performed was the presence of cells, yes/no, and the presence of microorganisms, yes/no.

Archived cytology slides from the cats included in the study were tracked and retrieved. For all cases where slides could be found, a second, blinded and standardized cytological evaluation was performed separately by two board-certified clinical pathologists (AH and JÖ). A protocol for the blinded evaluation was created to divide the cats into *Severe lipidosis* and *Other cytological findings*. The protocol is shown in Supplementary Files Table S3. The cytological findings evaluated were proportion of hepatocytes with vacuoles, degree of vacuolization, and presence of inflammatory cells and, if present, the number of inflammatory cells. The results from the re-evaluation were collected into a separate data sheet (Microsoft Excel). The results were compared between the two clinical pathologists, and in cases with inconsistent interpretation, slides were reviewed a second time by both clinical pathologists and discussed to gain agreement.

Cats were divided into two groups depending on results from liver cytology: *Severe lipidosis* and *Other cytological findings*. Inclusion in either group was based on results from the re-evaluation in the cats for which the archived slides had been found. For the remaining cats, information collected from the cytology report in the medical records was used. The *Severe lipidosis* group included cats with marked signs of hepatic lipidosis and no other cytological findings. For cats where re-evaluation had been performed, cats with >80% vacuolar changes in the hepatocyte’s cytoplasm in >80% of hepatocytes were included; for the remaining cats, information from the medical record generating similar cytological interpretations led to inclusion in the *Severe lipidosis* group. If no or mild to moderate vacuolation or other abnormalities were detected, the cats were included in the *Other cytological findings* group.

### 2.6. Statistical Methods

Statistical analyses were performed using a commercially available statistical software program (JMP Pro v. 16.0.0, Cary, NC, USA). Data were analyzed using descriptive as well as inferential statistics. Continuous variables were presented as median and interquartile range (IQR). The level of statistical significance was set at *p* < 0.05, if not otherwise indicated. Differences in proportions in categorical data (female/male, and yes or no regarding body weight reduction, feeding tube during hospital visit, vomiting, diarrhea, sampling from liver and/or bile, antibiotic treatment) were compared between the four clinical diagnosis categories and the cytological findings group using the Chi-squared and Fischer’s exact tests. Continuous data (age, body weight (BW), BW reduction, temperature, days of anorexia, laboratory variables, days of hospitalization) were compared between the four clinical diagnosis categories and between the cytological findings groups using the non-parametric Wilcoxon signed rank test. Uni- and multi-variable regression analyses were used to investigate the potential effects of case history (appetite, presence of anorexia, days of anorexia, presence of vomiting, and/or diarrhea), cat characteristics (age, sex, breed, body weight (BW), and BW reduction) and laboratory variables (hematology profile, ALT, ALP, GGT, albumin, protein, glucose, urea, creatinine, cholesterol, bilirubin, and bile acids) on SAA concentrations. The efficacy of the final model in the multivariable analysis to identify lipidosis was investigated by constructing receiver operating characteristic (ROC) curves and by calculating the area under the curve (AUC).

## 3. Results

### 3.1. Study Population

**Figure 1:**
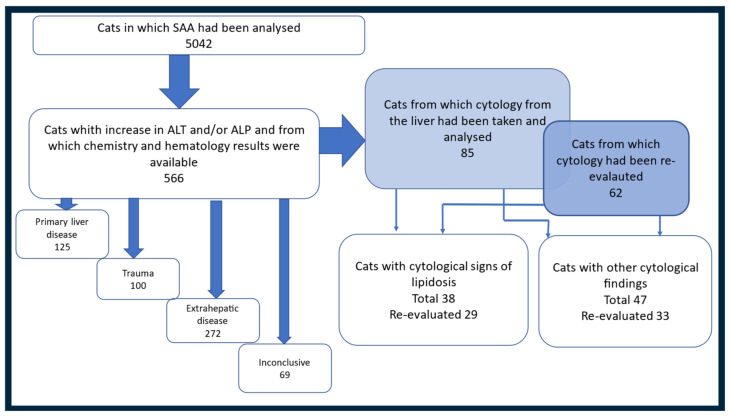
Flow chart showing the different steps in categorization of cats into clinical diagnosis categories and allocated into two groups based on cytology results. Number of cats in the different selection steps and in the different categories and groups are shown in the figure.

A flowchart of the different steps in the search process of the medical records and results are presented in Figure 1. During the period of interest, SAA was analyzed in 5042 cats. Blood activity of ALT and/or ALP was increased in 568 of these cats. Two cats were excluded from the study because the required chemistry panel was not analyzed at the same time point as analysis of SAA and liver enzymes. Accordingly, the final study population comprised 566 cats that fulfilled the inclusion criteria.

### 3.2. Comparison between Different Disease Categories

Grouping of the cats into four clinical diagnosis categories (Primary liver, Trauma, Extrahepatic, and Inconclusive) was based on diagnoses in the medical record system for 86% of the cats. For the remaining cats, diagnoses set after review of the medical records were used. One-hundred twenty-five cats were included in the Primary liver category, 100 cats were included in the Trauma category, 272 cats were included in the Extrahepatic category, and 69 cats were included in the Inconclusive category. Medical history, cat characteristic variables, sampling information, treatment options, and outcomes are shown in Table 1.

The results from hematology and SAA analyses and results from the chemistry analysis are presented in Table 2. Serum amyloid A was higher in the cats in the Trauma category compared to all other categories (*p* < 0.008). There was no difference in SAA between the other clinical diagnosis categories. Hemoglobin and hematocrit were higher in cats in the Primary liver disease and Trauma categories compared to cats in the other diagnosis categories. Cats in the Trauma category had lower total protein concentration compared to cats in the other categories.

### 3.3. Comparison between Groups Based on Cytology Findings

Fine-needle aspiration of the liver was performed in 94 of the 566 cats included in the study. In nine of these cats, cytology was non-diagnostic. Therefore, the cytology group included 85 cats (14.9% of the total study population). Cytology slides could be tracked and re-evaluated in 62 of these cats (10.8% of the total study population). For 23 cats, where slides were not available for re-evaluation, information from the cytology report in the medical record was used. Thirty-eight of the of cats examined with cytology were included in the Severe lipidosis group, and 47 cats were included in the Other cytological findings group; see Figure 1. Bile aspirates were performed in 54 (9.5%) of the cats included in the study, and microbiological culture in 48 (8.4%) of the cats. In the Severe lipidosis group, 76.3% of the cats had been treated with a feeding tube, compared to 29.8% of the cats in the Other cytological findings group (*p* < 0.0001). Medical history, cat characteristic variables, sampling information, and treatment options and outcomes for the cats evaluated with cytology are shown in Table 3.

**Figure 2:**
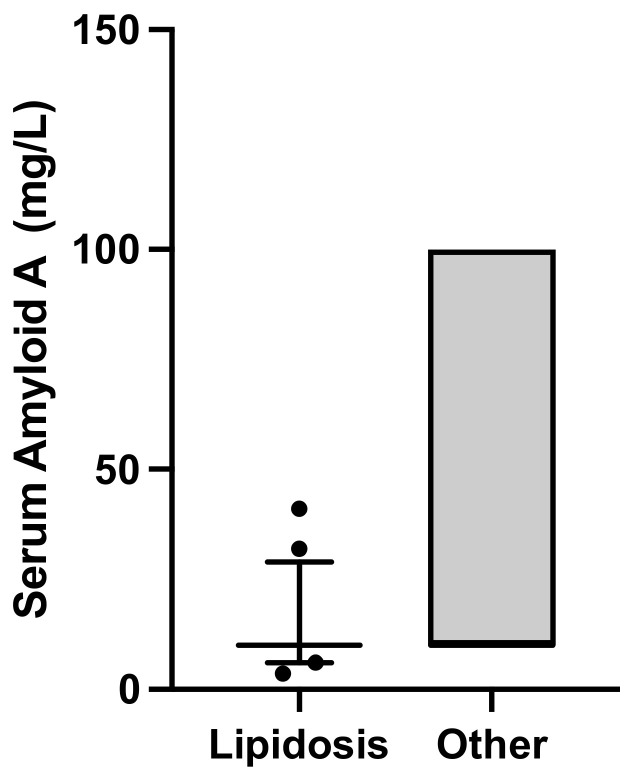
Box plot showing distribution of concentrations of SAA in 85 cats by cytology group, i.e., Severe lipidosis (n = 38) and Other cytological findings (n = 47) groups. The box shows interquartile range with median, and the whiskers show 5–95 percentiles. The median SAA in cats in the Severe lipidosis group was 10 mg/L (interquartile range 10–10), and the median SAA in cats in the group Other cytological findings was 10 mg/L (interquartile ranges 10–100), *p* < 0.0001.

**Figure 3:**
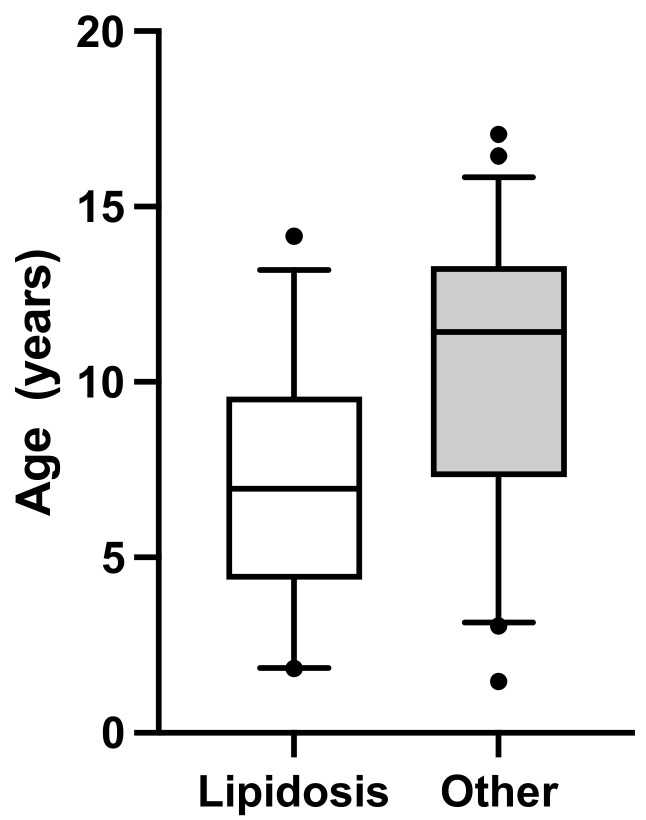
Box plot showing age distribution of 85 cats by cytology group, i.e., Severe lipidosis (n = 38) and Other cytological findings (n = 47) groups. The box shows interquartile range with median, and the whiskers show 5–95 percentiles. The median age in the Severe lipidosis group was 7.0 years (interquartile range 4.4–9.6), and the median age in the Other cytological findings group was 11.4 years (interquartile range 7.3–13.3), *p* = 0.0002.

Results from hematology and SAA analyses in the cats examined with cytology and results from chemistry analysis are presented in Table 4. Cats in the Severe lipidosis group had lower SAA concentration (*p* = 0.0001), see Figure 2; were younger (*p* = 0.0002), see Figure 3; and had lower albumin (*p* = 0.03) and urea (*p* = 0.02) serum concentrations than cats in the Other cytological findings group, see Table 4. Two cats in the Severe lipidosis group had a moderate increase in SAA concentration, 32 and 41 ug/mL, respectively. Serum amyloid A and age remained significant in the multiple regression analysis (*p* = 0.0001, R^2^ = 0.26).

**Figure 4:**
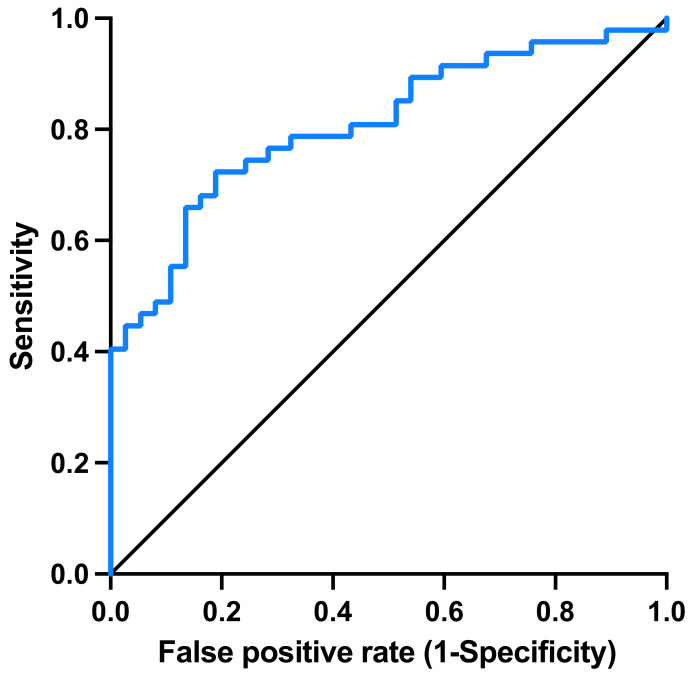
Receiver operating characteristic (ROC) curve for the final model, including age and SAA concentration, to identify presence of lipidosis (yes/no) in 85 cats. The area under the ROC curve (blue line) for the final model including SAA concentration and age was 0.81 (*p* < 0.001).

This final model had an AUC of 0.81 (*p* < 0.001) in identifying lipidosis; see Figure 4. The optimal cutoffs were an age of 11 years and a serum SAA concentration of <13 mg/L. The formula for calculating the probability value for the presence of severe lipidosis was Prob = 1/(1 + Exp (−2.46 + 0.19 × Age + 0.053 × SAA).

## 4. Discussion

Among cats with increased liver enzyme activity in a blood sample, SAA concentration was significantly higher in cats diagnosed with a traumatic disease compared to cats with other clinical diagnosis categories. Cats with a cytological diagnosis of severe hepatic lipidosis had lower SAA concentrations and were younger compared to cats with other cytological findings.

Diagnoses included in the trauma category were, for example, traffic injuries and falls from height, which often generate significant tissue trauma and, depending on location, a massive increase in liver enzyme activity due to hepatocyte damage. Tissue trauma is a well-known cause for inflammation, with an acute phase response and an appurtenant thereto increase in APPs [29,32]. Cats with a diagnosis of lameness were also included in the trauma category. Lameness can be observed in cats with septic arthritis and different types of wounds, all of which can generate systemic inflammation with an increase in APPs [25,32], as well as other non-inflammatory diseases. Even though significantly higher SAA concentration was demonstrated in cats in the trauma category compared to cats in the other clinical diagnosis categories, there was an overlap in SAA concentration between all diagnosis categories.

No difference in SAA concentration was detected between cats included in the *Primary liver*, *Extrahepatic*, and *Inconclusive* categories. Both *Primary liver* and *Extrahepatic* categories included cats with non-inflammatory disease, not suspected to have an increase in SAA, as well as cats with an inflammatory disease, with a potential to generate an increase in SAA. Examples of inflammatory diseases in cats included in the *Primary liver* category included hepatitis, cholangitis, and cholangiopathies [33]. The *Extrahepatic* category included cats with inflammatory diseases such as pyometra and pancreatitis. Pancreatitis and pyometra are inflammatory diseases known to have the capability to generate increased liver enzyme activities [34,35,36]. The most common non-inflammatory disease in cats included in the *Primary liver* category was hepatic lipidosis. Examples of non-inflammatory diseases in cats in the *Extrahepatic* category were diabetes mellitus and hyperthyroidism. Both diabetes mellitus and hyperthyroidism are diseases known to affect the liver and therefore generate an increase in liver enzymes [37,38,39,40].

In cats for which cytology of samples from the liver was performed, a significantly lower concentration of SAA was seen in cats with *Severe lipidosis* compared to cats with *Other cytological findings*. Hepatic lipidosis is a metabolic disturbance, generating accumulation of triglycerides stored in vacuoles in hepatocytes [9,41,42]. Minimal or no inflammation is seen in the liver tissue in cats with histopathological diagnoses of lipidosis [9,43]. An increase in APPs, such as SAA, is therefore not expected and has, to our knowledge, not previously been evaluated. In the group of cats with other cytological findings, cats with different etiologies for an increase in liver enzymes were included, but specific diagnoses were not set due to the retrospective nature of the study and because very few liver biopsies were taken (results not presented). This group may have included cats with inflammatory disease processes, such as hepatitis or cholangitis, with a potential to generate an increase in SAA. Only two cats in the *Severe lipidosis* group (total 38 cats) had a moderate increase in SAA; the remaining cats had SAA values under 10 mg/L, which is below previously reported cut-off points for determination of inflammation [29,44]. These two cats may have had hepatic lipidosis secondary to another inflammatory disease [9,42,45], but this could not be evaluated due to the retrospective nature of the study. Focal, localized, and even disseminated inflammatory pathological processes in the liver, generating an increase in SAA, could also have been missed with cytology in these cats. Nodular, focal, or other processes localized around portal regions have been shown to be missed in samples from cats diagnosed by cytology as having hepatic lipidosis when results from cytology were compared to histopathology [46]. Reduced SAA synthesis due to decreased liver function in cats with severe lipidosis may also have contributed to the detected difference in SAA concentration between the two cytology groups. Serum concentrations of albumin and urea were lower in the *Severe lipidosis* group in the univariable analysis but did not remain significant in the final multivariable regression model. Both albumin and urea are synthesized in the liver, and their concentrations may decrease in patients with severe hepatic failure. Glucose, also a marker for hepatic function, did not differ between the cytology groups, and the lower concentrations of albumin and urea may be explained by several other causes. There were no significant differences in other inflammatory markers, such as hematology or proteins, between the two groups.

Cats in the *Severe lipidosis* group were younger compared to cats in the *Other cytological findings* group. The optimal cut-off for identifying severe lipidosis in this group of cats was an age of 11 years and an SAA concentration of 13 mg/L. Hepatic lipidosis can be seen in cats of any age but is mainly reported in middle-aged cats [6,42]. In one study of 77 cats with hepatic lipidosis, the median age of affected cats was eight years (range one to sixteen years), and ten cats were less than four years old [4].

Forty-five percent of cats that had liver cytology performed were diagnosed with hepatic lipidosis. This is a similar number of cats as in a previous study, where 49% (n = 175) of cats presenting with a case history and clinical signs indicative of liver disease had hepatic lipidosis based on histopathology [43]. Histopathologic examination of liver tissue is often recommended to establish a specific diagnosis, and discrepancy between histology and cytology of the liver in dogs and cats has been reported [10,12,46]. However, in widespread and diffuse pathological changes, such as in severe hepatic lipidosis, cytology has an acceptable agreement with histopathology and may be used as a reliable diagnostic tool [8,47,48]. Full agreement between cytology and histopathology for vacuolar hepatopathy was reported in 15/18 cats in a previous publication [49]. In that study, seven cats were diagnosed with vacuolar hepatopathy by cytology, but additional pathological changes were detected with histopathology, indicating that cytology has a high sensitivity to detect hepatic lipidosis but lower sensitivity to detect other pathologies in the liver [49]. In our study, only cats with cytological findings of marked vacuolar changes were included in the lipidosis group. Cats with cytology findings of less severe vacuolar changes, indicating moderate hepatic lipidosis, were included in the *Other cytological findings* group. This was done because some of these cats might have had other pathologies undetected by cytology and because we sought to increase the specificity of a hepatic lipidosis diagnosis. Consequently, cats with moderate lipidosis, and possibly no other pathologies, could have been included in the other cytological findings group. To increase accuracy in diagnosing cats with cytology, a standardized blinded cytological evaluation was performed independently by two clinical pathologists. To further increase the accuracy, special staining, for example, by using Sudan Oil, could have been used, but that was not done due to the retrospective nature of the study. Fine-needle aspirates from the liver were performed in more than 67% of the cats included in the category of cats with probable primary liver disease. This high number of cytological examinations, compared to histopathology evaluation, is probably due to the possibility of a rapid in-house cytological examination at the animal hospital. The less invasive sampling technique for cytology may also increase the willingness of the attending veterinarian and cat owner to undertake cytology sampling.

A limitation of this study, due to its retrospective nature, was that chemistry analyses were performed using different methods on two different instruments, depending on time for sampling and analysis. Numerical values could therefore not be used, and deviations in percentage from respective reference values for the different instruments were used instead. Another limitation, also due to the study’s retrospective nature, was that diagnoses used to group cats with increase in liver enzymes into four clinical disease categories were set by several different veterinarians with different clinical experience. Diagnoses were sometimes not determined, and an inconclusive group was created to enable usage of data from this relatively large group of cats. To increase the likelihood of a correct diagnosis, the medical records were thoroughly reevaluated by two experienced board-certified specialists for the cats where specific diagnoses were missing. Finally, the final statistical model for identifying lipidosis should be tested prospectively in cats.

## 5. Conclusions

In cats with increased liver enzymes, cats with a history of trauma had higher SAA concentrations compared to cats in the other clinical diagnosis categories. Cats with cytological findings suggesting severe hepatic lipidosis had significantly lower serum SAA concentration and were younger compared to cats with other cytological findings in the liver. Based on the findings from this retrospective study, for cats presenting with increased liver enzyme activity due to a potential diagnosis of primary liver disease, a diagnosis of hepatic lipidosis is more likely, compared to inflammatory liver disease, if the SAA concentration is low and the cat is younger than 11 years.

## Figures and Tables

**Table 1:** Summary of cat characteristics, diagnostic procedures, and outcomes in 566 cats by four clinical disease categories. Results are reported as median and interquartile range. Results in percentage represent number of cats that were sampled or treated (y). Within each row, values with the same superscript letter (a–c) did not differ significantly after Bonferroni correction (*p* < 0.008).

Variables	Primary Liver Disease (n = 125)	Trauma (n = 100)	Extrahepatic Disease (n = 274)	Non-Conclusive (n = 69)
Age (y)	9.5 (6.1–12.6) ^a^	2.6 (1.0–5.6) ^b^	11.2 (6.8–14.2) ^ac^	12.2 (8.4–17.0) ^c^
Sex (f/m)	64/61 ^a^	52/48 ^a^	129/143 ^a^	40/29 ^a^
BW (kg)	4.2 (3.4–4.9) ^a^	4.0 (3.2–4.8) ^a^	3.9 (3.2–4.8) ^a^	3.9 (3.3–4.8) ^a^
BW reduction (y/n) (%)	55/69 ^a^	0/100 ^b^	66/206 ^c^	33/36 ^ad^
	44.3	0	24.3	47.8
Days of anorexia (d)	3 (2–7) ^a^	0 (0–0) ^b^	0 (0–2) ^c^	1 (0–3) ^c^
Vomiting (y/n) (% y)	56/69 ^a^	1/99 ^b^	127/145 ^c^	28/4 ^ac^
	44.8	1.0	46.3	40.6
Diarrhea (y/n) (% y)	4/121 ^a^	1/99 ^a^	23/249 ^a^	4/65 ^a^
	3.2	1.0	8.5	5.8
Body temperature (°C)	38.3 (38.0–38.8) ^a^	38.5(37.8–39.2.) ^a^	38.2 (37.6–38.7) ^a^	38.1 (37.7–39.0) ^a^
Feeding tube (y/n) (% y)	54/70 ^a^	5/95 ^ab^	10/262 ^b^	3/66 ^b^
	43.5	5.0	3.7	4.3
FNA liver (y/n) (% y)	85/40 ^a^	0/100 ^b^	6/266 ^b^	1/69 ^b^
	67.2	0	2.2	1.4
Bile aspiration (y/n) (% y)	52/73 ^a^	0/100 ^b^	2/270 ^b^	1/68 ^b^
	40.8	0	0.7	1.4
Culture liver (y/n) (% y)	13/111 ^a^	0/100 ^b^	0/272 ^b^	1/68 ^b^
	10.5	0	0	0
Culture bile (y/n) (% y)	51/74 ^a^	0/100 ^b^	2/270 ^b^	1/67 ^b^
	40.8	0	0.7	1.5
Antibiotic treatment (y/n) (% y)	70/54 ^a^	32/68 ^b^	47/225 ^c^	7/6 ^c^
	56.4	32.0	17.3	11.4
Hospitalization days (d)	3 (2–5) ^a^	2 (1–3) ^b^	1 (0–2) ^c^	0 (0–2) ^c^

**Table 2:** Summary of hematology and chemistry results and serum SAA concentrations in 566 cats by four clinical disease categories. For chemistry results, number of cats are reported as above (A), within (W), and below (B) the normal reference range in and proportions are expressed as percentages within brackets. Values are reported as median and interquartile ranges (IQRs). Within each row, values with the same superscript letter (a,b) did not differ significantly after Bonferroni correction (*p* < 0.008).

Variable	Primary Liver (n = 125)	Trauma (n = 100)	Extrahepatic (n = 272)	Inconclusive (n = 69)
Hct (%) (n = 566)	36 (30–40) ^a^	35 (30–40) ^a^	40 (35–45) ^b^	37 (32–45) ^ab^
Hb (g/L) (n = 566)	120 (108–135) ^a^	119 (109–140) ^a^	134 (119–150) ^b^	130 (110–154) ^ab^
WBC (×10^9^/L) (n = 566)	11.2 (7.4–17.3) ^ab^	12.4 (9.3–18.2) ^a^	9.5 (6.6–14.2) ^b^	10.4 (7.2–16.2) ^ab^
Segmented neutrophils (×10^9^/L) (n = 317)	9.0 (4.6–12.9) ^a^	11.3 (5.6–15.2) ^a^	7.4 (4.4–12.8) ^a^	8.4 (5.0–10.9) ^a^
Band neutrophils (×10^9^/L) (n = 317)	0.0 (0–0.3) ^a^	0.2 (0–1.6) ^a^	0.0 (0–0.2) ^a^	0.0 (0–0.2) ^a^
Toxicity (y/n) y% (n = 42)	17/108 ^a^	3/97 ^a^	19/255 ^a^	2/67 ^a^
	13.6	3.0	6.9	2.9
Monocytes (×10^9^/L) (n = 317)	0.3 (0.1–0.6) ^ab^	0.2 (0.1–0.4) ^a^	0.3 (0.1–0.6) ^ab^	0.4 (0.2–0.6) ^b^
Lymphocytes (×10^9^/L) (n = 317)	1.2 (0.7–2.0) ^a^	1.5 (1.0–2.7) ^a^	1.3 (0.6–2.1) ^a^	1.6 (0.8–2.6) ^a^
Eosinophils (×10^9^/L) (n = 317)	0.2 (0–0.4) ^a^	0.15 (0–0.3) ^a^	0.2 (0–0.4) ^a^	0.0 (0–0.5) ^a^
Basophils (×10^9^/L) (n = 317)	0.0 (0.0–0.0) ^a^	0.0 (0.0–0.0) ^a^	0.0 (0.0–0.0) ^a^	0.0 (0.0–0.0) ^a^
Thrombocytes (×10^9^/L) (n = 566)	275 (171–356) ^a^	239 (179–318) ^a^	271 (175–351) ^a^	261 (199–346) ^a^
SAA (ug/mL) (n = 566)	10 (10–68) ^a^	72 (10–100) ^b^	10 (10–71) ^a^	10 (10–79) ^a^
ALT (A/W) (%) (n = 561)	121/3 (98/2) ^a^	100/0 (100/0) ^a^	261/11 (96/4) ^a^	63/2 (97/3) ^a^
ALP (A/W) (%) (n = 337)	82/27 (75/25) ^a^	7/19 (27/73) ^b^	60/92 (39/61) ^b^	37/13 (74/26) ^a^
GGT (A/W) (%) (n = 321)	14/85 (14/86) ^a^	0/24 (0/100) ^b^	6/144 (4/96) ^b^	3/45 (6/94) ^b^
Albumin (A/W/B)(%) (n = 566)	5/109/11 (4/87/9) ^a^	4/91/5 (4/91/5) ^a^	29/227/16 (11/84/6) ^b^	2/67/0 (3/97/0) ^a,b^
Total protein (A/W/B) (%) (n = 566)	19/98/8 (15/78/6) ^a^	3/84/13 (3/84/13) ^b^	53/208/11 (19/77/4) ^a^	10/58/1 (15/84/1) ^a^
Urea (A/W/B) (%) (n = 562)	15/101/7 (12/82/6) ^a^	13/87/0 (13/87/0) ^a^	65/206/0 (24/76/0) ^b^	12/53/0 (18/78/4) ^a,b^
Cholesterol (A/W) (%) (n = 315)	83/16 (84/16/) ^a^	21/3 (88/12) ^a,b^	138/10 (93/7) ^b^	35/9 (80/20) ^a^
Glucose (A/W/B) (%) (n = 563)	47/77/0 (38/62/0) ^a^	54/46/0 (54/46/0) ^b^	100/169/1 (37/62/1) ^a^	26/42/11 (38/61/1) ^a,b^
Creatinine (A/W) (%) (n = 563)	17/105 (14/86) ^a^	10/90 (10/90) ^a^	61/211 (22/78) ^b^	8/62 (12/88) ^a,b^

**Table 3:** Summary of cat characteristics, diagnostic procedures, and outcome in 85 cats by Severe lipidosis and Other cytological findings groups, based on cytology results from the liver. Results for continuous data are reported as median and interquartile range. For binary outcome variables (yes/no), results are reported as number and percentage within brackets. Within each row, values with the same superscript letter (a,b) did not differ significantly (*p* < 0.05).

Variables	Severe Lipidosis (n = 38)	Other Cytological Findings (n = 47)
Age (years) (n = 85)	6.9 (4.4–9.6) ^a^	11.4 (4.3–13.3) ^b^
Sex (female/male) (n = 85)	24/14 ^a^	18/29 ^a^
Body weight (BW) (kg) (n = 85)	4.1 (3.4–4.9) ^a^	4.5 (3.7–5.0) ^a^
BW reduction (y/n) (%) (n = 85)	19/19 (50/50) ^a^	19/30 (36/64) ^a^
Days of anorexia (days) (n = 73)	3 (3–10) ^a^	3 (1–7) ^a^
Vomiting (y/n) (%) (n = 85)	12/26 (32/68) ^a^	19/28 (40/60) ^a^
Diarrhea (y/n) (%) (n = 85)	1/37 (3/86) ^a^	2/45 (4/96) ^a^
Body temperature (°C) (n = 75)	38.3 (38.1–38.7) ^a^	38.3 (38.0–39.0) ^a^
Feeding tube (y/n) (%) (n = 85)	29/9 (76/24) ^a^	14/33 (30/70) ^a^
Culture liver (y/n) (%) (n = 84)	5/33 (13/87) ^a^	5/41 (10/90) ^a^
Culture bile (y/n) (%) (n = 85)	19/19 (50/50) ^a^	25/22 (53/47) ^a^
Antibiotic treatment (y/n) (%) (n = 84)	24/14 (63/27) ^a^	23/23 (50/50) ^a^
Hospitalization (days) (n = 85)	4 (3–8) ^a^	3.0 (1–4) ^b^

**Table 4:** Summary of hematology and chemistry results and SAA concentration in 85 cats evaluated withcytology from the liver, divided into Severe lipidosis and Other cytological findings groups. The number of cats with results available are reported within brackets. For chemistry results number of cats are reported as above (A), within (W), and below (B) the normal reference range, and proportions are expressed as percentages within brackets Within each row, values with the same superscript letter (a,b) did not differ significantly (*p* < 0.05).

Variable	Severe Hepatic Lipidosis (n = 38)	Other Cytological Findings (n = 47)
Hct (%) (n = 84)	35 (29–38) ^a^	36 (31–41) ^a^
Hb (g/L) (n = 84)	117 (95–134) ^a^	121 (109–136) ^a^
WBC (×10^9^/L) (n = 84)	9.8 (5.9–13.1) ^a^	10.4 (7.7–14.2) ^a^
Segmented neutrophils (×10^9^/L) (n = 67)	7.9 (3.6–11.3) ^a^	8.1 (4.0–12.2) ^a^
Band neutrophils (×10^9^/L) (n = 67)	0.0 (0–0.1) ^a^	0.1 (0–0.3) ^a^
Monocytes (×10^9^/L) (n = 67)	0.3 (0.1–0.5) ^a^	0.3 (0.1–0.5) ^a^
Lymphocytes (×10^9^/L) (n = 67)	1.3 (0.9–2.2) ^a^	1.0 (0.6–1.7) ^a^
Eosinophils (×10^9^/L) (n = 67)	0.2 (0.0–1-0.4) ^a^	0.3 (0–0.4) ^a^
Basophils (×10^9^/L) (n = 67)	0.0 (0.0–0.0) ^a^	0.0 (0.0–0.05) ^a^
Thrombocytes (×10^9^/L) (n = 85)	280 (177–382) ^a^	255 (158–325) ^a^
SAA (ug/mL) (n = 85)	10 (10–10) ^a^	10 (10–100) ^b^
ALT (A/W) (n = 84)	35/2 (95/5) ^a^	47/0 (100/0) ^a^
ALP (A/W) (n = 73)	33/1 (97/3) ^a^	26/13 (67/33) ^a^
GGT (A/W) (n = 66)	26/4 (87/13) ^a^	33/3 (92/8) ^a^
Albumin (A/W/B) (n = 85)	4/31/3 (10/82/8) ^a^	0/46/1 (0/98/2) ^a^
Total protein (A/W/B) (n = 85)	3/33/2 (8/87/5) ^b^	7/40/0 (15/85/0) ^a^
Urea (A/W/B) (n = 83)	0/32/5 (0/86/16) ^b^	5/40/1 (11/87/2) ^a^
Cholesterol (A/W) (n = 65)	25/8 (76/24) ^a^	27/5 (84/16) ^a^
Glucose (A/W/B) (n = 84)	14/24/0 (37/63/0) ^a^	17/29/0 (37/53/0) ^a^
Creatinine (A/W) (n = 83)	3/34 (8/92) ^a^	6/40 (13/87) ^a^

## Data Availability

The data presented in this study are available within this article and Supplementary Materials.

## Supplementary Materials

The following supporting information can be downloaded at: https://www.mdpi.com/article/10.3390/vetsci11070298/s1, Table S1: Examples of diagnoses in the diagnostic index coding system, used for inclusion in four diagnosis-based categories; Table S2: Standardized protocol used for blinded cytology evaluation; Table S3: Reference ranges for the chemistry instruments used in the study.

## References

[1] Alvarez L., Whittemore J. (2009). Liver enzyme elevations in dogs: Diagnostic approach. Compend. Contin. Educ. Vet..

[2] Center S.A. (2007). Interpretation of liver enzymes. Vet. Clin. N. Am. Small Anim. Pract..

[3] Brown B., Mauldin G.E., Armstrong J., Moroff S.D., Mauldin G.N. (2000). Metabolic and hormonal alterations in cats with hepatic lipidosis. J. Vet. Intern. Med..

[4] Center S.A., Crawford M.A., Guida L., Erb H.N., King J.A. (1993). Retrospective study of 77 cats with severe hepatic lipidosis: 1975–1990. J. Vet. Intern. Med..

[5] Brain P.H., Barrs V.R., Martin P., Baral R., White J.D., Beatty J.A. (2006). Feline cholecystitis and acute neutrophilic cholangitis: Clinical findings, bacterial isolates and response to treatment in six cases. J. Feline Med. Surg..

[6] Center S.A. (2005). Feline hepatic lipidosis. Vet. Clin. N. Am. Small Anim. Pract..

[7] Day D.G. (1995). Feline cholangiohepatitis complex. Vet. Clin. N. Am. Small Anim. Pract..

[8] Roth L. (2001). Comparison of liver cytology and biopsy diagnoses in dogs and cats: 56 cases. Vet. Clin. Pathol..

[9] Zawie D.A., Garvey M.S. (1984). Feline hepatic disease. Vet. Clin. N. Am. Small Anim. Pract..

[10] Kerwin S.C. (1995). Hepatic aspiration and biopsy techniques. Vet. Clin. N. Am. Small Anim. Pract..

[11] Rothuizen J., Twedt D.C. (2009). Liver biopsy techniques. Vet. Clin. N. Am. Small Anim. Pract..

[12] Cole T.L., Center S.A., Flood S.N. (2002). Diagnostic comparison of needle and wedge biopsy specimens of the liver in dogs and cats. J. Am. Vet. Med. Assoc..

[13] Kemp S.D., Zimmerman K.L., Panciera D.L., Monroe W.E., Leib M.S., Lanz O.I. (2015). A comparison of liver sampling techniques in dogs. J. Vet. Intern. Med..

[14] Boland L., Beatty J. (2017). Feline Cholangitis. Vet. Clin. N. Am. Small Anim. Pract..

[15] Hughes D., King L.G. (1995). The diagnosis and management of acute liver failure in dogs and cats. Vet. Clin. N. Am. Small Anim. Pract..

[16] Lisman T., Leebeek F.W. (2007). Hemostatic alterations in liver disease: A review on pathophysiology, clinical consequences, and treatment. Dig. Surg..

[17] Kavanagh C., Shaw S., Webster C.R. (2011). Coagulation in hepatobiliary disease. J. Vet. Emerg. Crit. Care.

[18] Reece J., Pavlick M., Penninck D.G., Webster C.R.L. (2020). Hemorrhage and complications associated with percutaneous ultrasound guided liver biopsy in dogs. J. Vet. Intern. Med..

[19] Pavlick M., Webster C.R., Penninck D.G. (2019). Bleeding risk and complications associated with percutaneous ultrasound-guided liver biopsy in cats. J. Feline Med. Surg..

[20] Moritz A.K., Köhler C., Fromme V., Winter K., Alef M., Kiefer I. (2018). Complications of ultrasound-guided liver biopsies in dogs and cats. Tierarztl Prax Ausg K Kleintiere Heimtiere.

[21] Bigge L.A., Brown D.J., Penninck D.G. (2001). Correlation between coagulation profile findings and bleeding complications after ultrasound-guided biopsies: 434 cases (1993–1996). J. Am. Anim. Hosp. Assoc..

[22] Balkman C. (2009). Hepatobiliary neoplasia in dogs and cats. Vet Clin. N. Am. Small Anim. Pract..

[23] Ceron J.J., Eckersall P.D., Martýnez-Subiela S. (2005). Acute phase proteins in dogs and cats: Current knowledge and future perspectives. Vet. Clin. Pathol..

[24] Kajikawa T., Furuta A., Onishi T., Tajima T., Sugii S. (1999). Changes in concentrations of serum amyloid A protein, alpha 1-acid glycoprotein, haptoglobin, and C-reactive protein in feline sera due to induced inflammation and surgery. Vet. Immunol. Immunopathol..

[25] Paltrinieri S. (2008). The feline acute phase reaction. Vet. J..

[26] Sasaki K., Ma Z., Khatlani T.S., Okuda M., Inokuma H., Onishi T. (2003). Evaluation of feline serum amyloid A (SAA) as an inflammatory marker. J. Vet. Med. Sci..

[27] Christensen M., Jacobsen S., Ichiyanagi T., Kjelgaard-Hansen M. (2012). Evaluation of an automated assay based on monoclonal anti-human serum amyloid A (SAA) antibodies for measurement of canine, feline, and equine SAA. Vet. J..

[28] Kann R.K., Seddon J.M., Henning J., Meers J. (2012). Acute phase proteins in healthy and sick cats. Res. Vet. Sci..

[29] Tamamoto T., Ohno K., Takahashi M., Nakashima K., Fujino Y., Tsujimoto H. (2013). Serum amyloid A as a prognostic marker in cats with various diseases. J. Vet. Diagn. Investig. Off. Publ. Am. Assoc. Vet. Lab. Diagn..

[30] Stockham S. (2008). Fundamentals of Veterinary Clinical Pathology.

[31] Hansen A.E., Schaap M.K., Kjelgaard-Hansen M. (2006). Evaluation of a commercially available human serum amyloid A (SAA) turbidimetric immunoassay for determination of feline SAA concentration. Vet. Res. Commun..

[32] Troìa R., Gruarin M., Foglia A., Agnoli C., Dondi F., Giunti M. (2017). Serum amyloid A in the diagnosis of feline sepsis. J. Vet. Diagn. Investig. Off. Publ. Am. Assoc. Vet. Lab. Diagn..

[33] Yuki M., Aoyama R., Nakagawa M., Hirano T., Naitoh E., Kainuma D. (2020). A Clinical Investigation on Serum Amyloid A Concentration in Client-Owned Healthy and Diseased Cats in a Primary Care Animal Hospital. Vet. Sci..

[34] Černá P., Kilpatrick S., Gunn-Moore D.A. (2020). Feline comorbidities: What do we really know about feline triaditis?. J. Feline Med. Surg..

[35] Fragkou F.C., Adamama-Moraitou K.K., Poutahidis T., Prassinos N.N., Kritsepi-Konstantinou M., Xenoulis P.G., Steiner J.M., Lidbury J.A., Suchodolski J.S., Rallis T.S. (2016). Prevalence and Clinicopathological Features of Triaditis in a Prospective Case Series of Symptomatic and Asymptomatic Cats. J. Vet. Intern. Med..

[36] Hagman R. (2018). Pyometra in Small Animals. Vet. Clin. N. Am. Small Anim. Pract..

[37] Campbell J., Chapman P., Klag A. (2022). The Prevalence, Magnitude, and Reversibility of Elevated Liver Enzyme Activities in Hyperthyroid Cats Presenting for Iodine-131 Treatment. Front. Vet. Sci..

[38] Papakonstantinou S. (2023). Assessment of serum glutamate dehydrogenase activity and comparison to serum alanine aminotransferase activity in cats with increased blood total T4 concentration. J. Vet. Diagn. Investig. Off. Publ. Am. Assoc. Vet. Lab. Diagn..

[39] Schaer M., Ginn P.E. (1999). Iatrogenic Cushing’s syndrome and steroid hepatopathy in a cat. J. Am. Anim. Hosp. Assoc..

[40] Crenshaw K.L., Peterson M.E. (1996). Pretreatment clinical and laboratory evaluation of cats with diabetes mellitus: 104 cases (1992–1994). J. Am. Vet. Med. Assoc..

[41] Mazaki-Tovi M., Abood S.K., Segev G., Schenck P.A. (2013). Alterations in adipokines in feline hepatic lipidosis. J. Vet. Intern. Med..

[42] Valtolina C., Favier R.P. (2017). Feline Hepatic Lipidosis. Vet. Clin. N. Am. Small Anim. Pract..

[43] Gagne J.M., Weiss D.J., Armstrong P.J. (1996). Histopathologic evaluation of feline inflammatory liver disease. Vet. Pathol..

[44] Waugh E.M., Haining H., Harvie J., Ridyard A.E., Eckersall P.D. (2022). Validation of an automated immunoturbidimetric assay for feline serum amyloid A. BMC Vet. Res..

[45] Jacobs G., Cornelius L., Allen S., Greene C. (1989). Treatment of idiopathic hepatic lipidosis in cats: 11 cases (1986–1987). J. Am. Vet. Med. Assoc..

[46] Willard M.D., Weeks B.R., Johnson M. (1999). Fine-needle aspirate cytology suggesting hepatic lipidosis in four cats with infiltrative hepatic disease. J. Feline Med. Surg..

[47] Weiss D.J., Blauvelt M., Aird B. (2001). Cytologic evaluation of inflammation in canine liver aspirates. Vet. Clin. Pathol..

[48] Chaivoravitsakul N., Chankow K., Horoongruang K., Limpongsai L., Tantarawanich A., Pluemhathaikij L., Rattanapinyopituk K., Angkanaporn K. (2021). Comparison of fine-needle cytologic diagnosis between the left and right liver lobes of dogs and cats with diffuse liver disease. Vet. World.

[49] Wang K.Y., Panciera D.L., Al-Rukibat R.K., Radi Z.A. (2004). Accuracy of ultrasound-guided fine-needle aspiration of the liver and cytologic findings in dogs and cats: 97 cases (1990–2000). J. Am. Vet. Med. Assoc..

