# Supplementary material for: The Diagnostic Value of Serum Amyloid A and Other Laboratory and Clinical Variables in Cats with Increased Liver Enzyme Activity
Source: Vet Sci. 2024 Jul 1;11(7):298. doi: 10.3390/vetsci11070298 (PMC11281469; doi:10.3390/vetsci11070298)
Supplement: Supplementary file 1 [file vetsci-11-00298-s001.zip › vetsci-3041211-supplementary.pdf]

Supplementary Files Table S1. Reference ranges for cats for chemistry analytes used at AniCura Bagarmossen Small Animal Hospital Laboratory.

**Cobas c311**		
**Analyte**	**Values**	**Unit**
Albumin	27-37	g/L
ALP	<1.0	ukat/L
ALT	< 1.2	ukat/L
Cholesterol	1.8-5.1	mmol/L
Creatinine	<160	umol/L
Glucose	3.4-8.5	mmol/L
SAA	<10.0	mg/L
Total Protein	57-80	g/L
Urea	4.0-13.0	mmol/L
**Catalyst**		
**Analyte**	**Values**	**Unit**
Albumin	22-40	g/L
ALP	14-111	U/L
ALT	12-130	U/L
Cholesterol	1.58-5.81	umol/L
Creatinine	71-212	umol/L
Glucose	4.11-8.84	mmol/L
Total Protein	57-89	g/L
Urea	5.7-12.9	mol/L

Supplementary Files Table S2. Examples of diagnoses used to categorize cats with increased liver enzymes into four categories based on suspected cause of increased liver enzyme activity.

	**Examples of diagnoses in the diagnostic index coding system, used for inclusion in four diagnosis-based categories**
**Primary liver**	Lipidosis<br>Cholangial hepatitis<br>Hepatitis<br>Biliary stones<br>Hepatic neoplasia<br>Portosystemic shunts<br>Icterus
**Trauma**	Trauma, unspecified<br>Traffic injuries<br>Fall from heights<br>Fractures<br>Luxations/subluxations<br>Lameness
**Extrahepatic**	Diabetes mellitus<br>Diabetic ketoacidosis<br>Hyperthyroidism<br>Pancreatitis<br>Anemia with icterus<br>Foreign body gastrointestinal system<br>Intoxication<br>Feline infectious peritonitis<br>Pyometra<br>Urinary tract disease<br>Hypertrophic cardiomyopathy
**Inconclusive**	Anorexia (unspecified and no clear cause)<br>Euthanasia (and no more information available)<br>Seizure (unexplained)<br>Alopecia<br>Itching<br>Epistaxis<br>Clinical signs liver unspecified

Supplementary Files Table S3. Standardized protocol used for blinded cytology evaluation. A grading system with four groups was created. Cats with cytological findings that met any of the criteria for grades 1–3 were all included in the *Other cytological findings* group. Cats with cytological findings consistent with grade 4 for all parameters were included in the *Severe lipidosis* group. The protocol was used by two blinded clinical pathologists for the re-evaluation in cats where cytology slides could be tracked. If there was a disagreement in any scoring (cats classified into different groups), the slides were re-evaluated a second time and discussed to meet an agreement.

	**Other cytological findings**			**Severe lipidosis**
Grade	**1**	**2**	**3**	**4**
Proportion of hepatocytes with vacuoles	0-80%	6-25%	26-80%	81-100%
Amount of vacuoles in cytoplasm	0-25%	26-50%	51-75%	76-100%
Number of neutrophils close to or between hepatocytes	Many	Moderate	Few	None or occasionally
Lymphocytes	Many	Moderate	Few	None or occasionally
Macrophages	Moderate	Few	None or occasionally	None or occasionally
Mast cells	Moderate	Few	None or occasionally	None or occasionally
Plasma cells	moderate	Few	None or occasionally	None or occasionally
Atypical cells	Present	Present	Present	Absent
